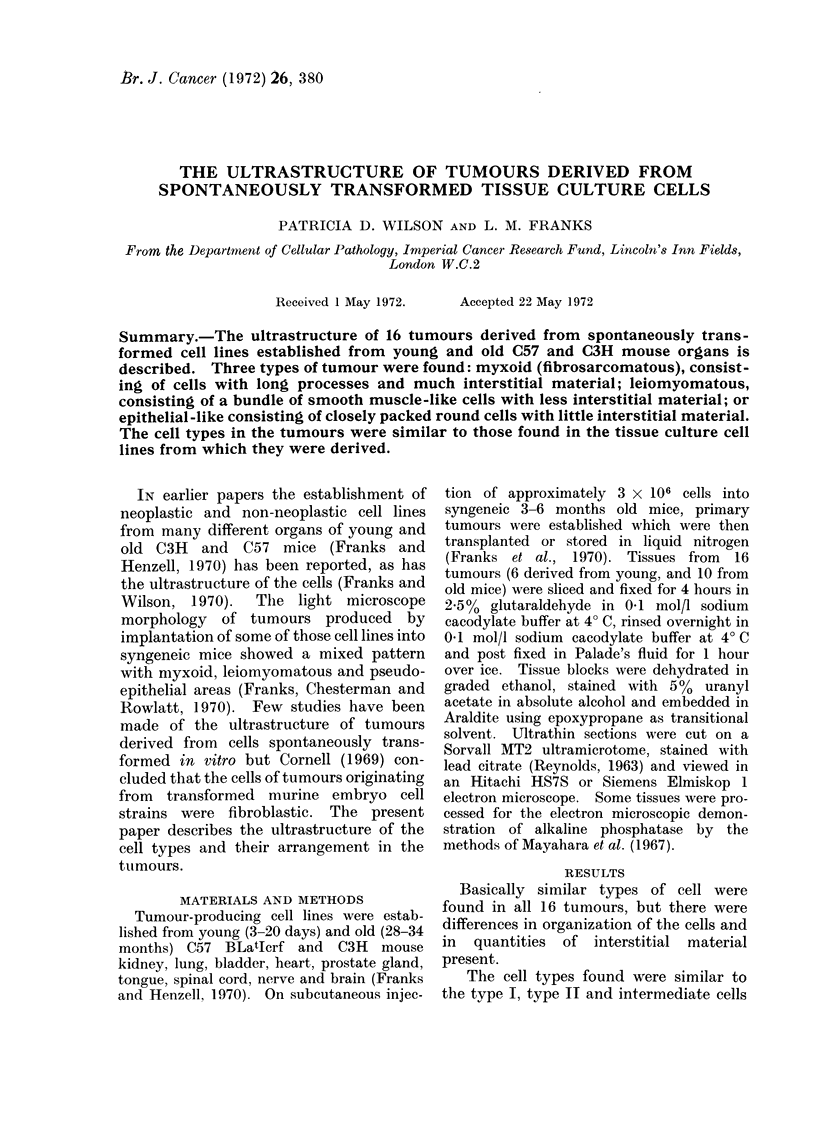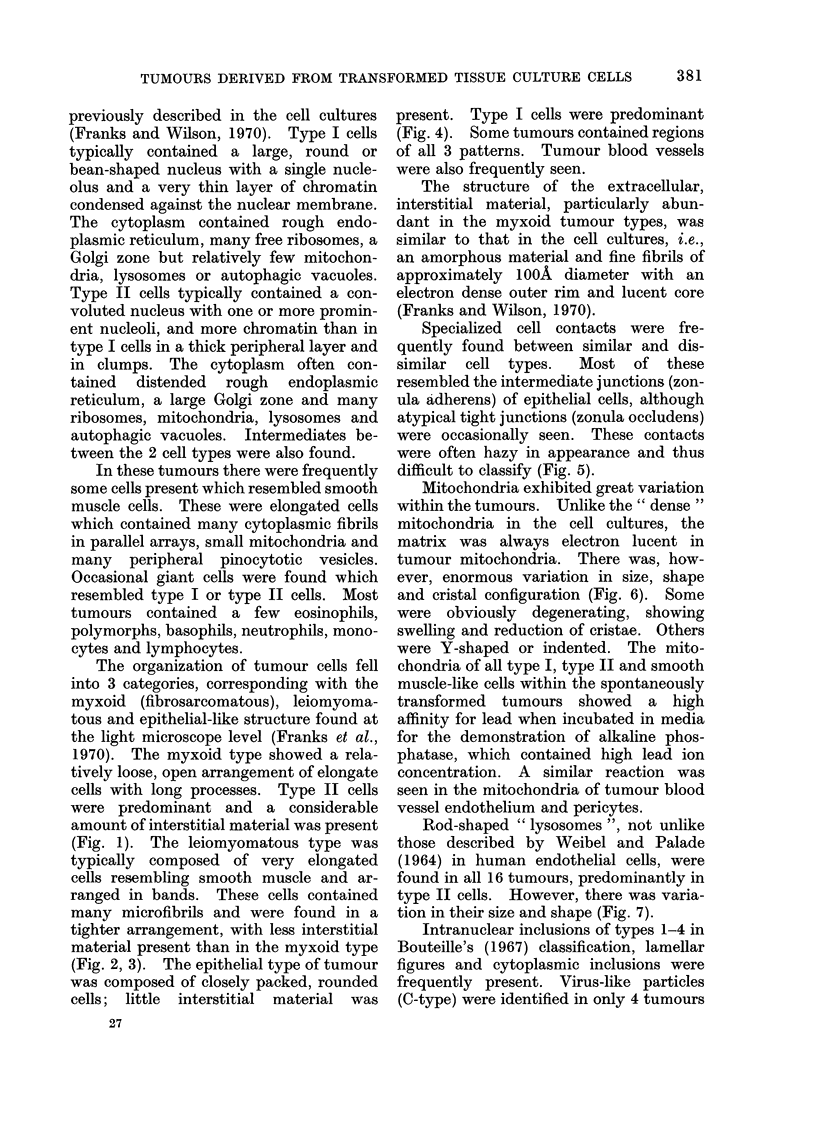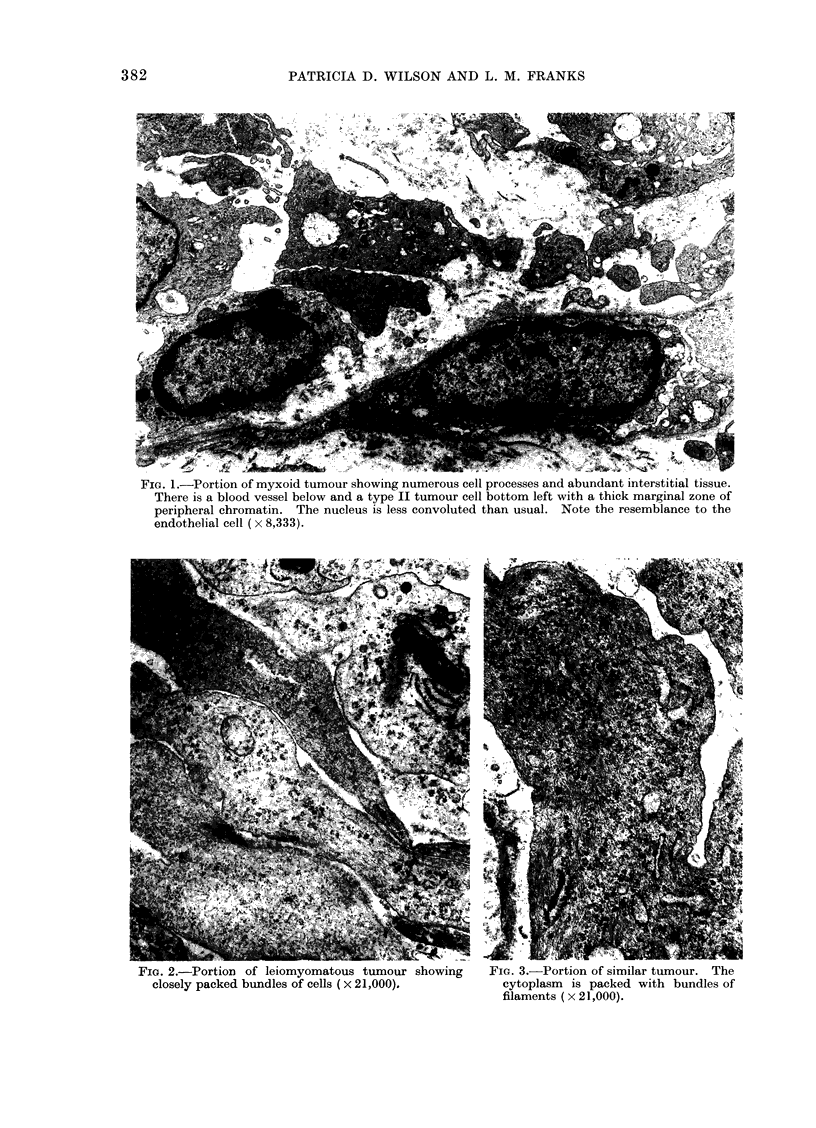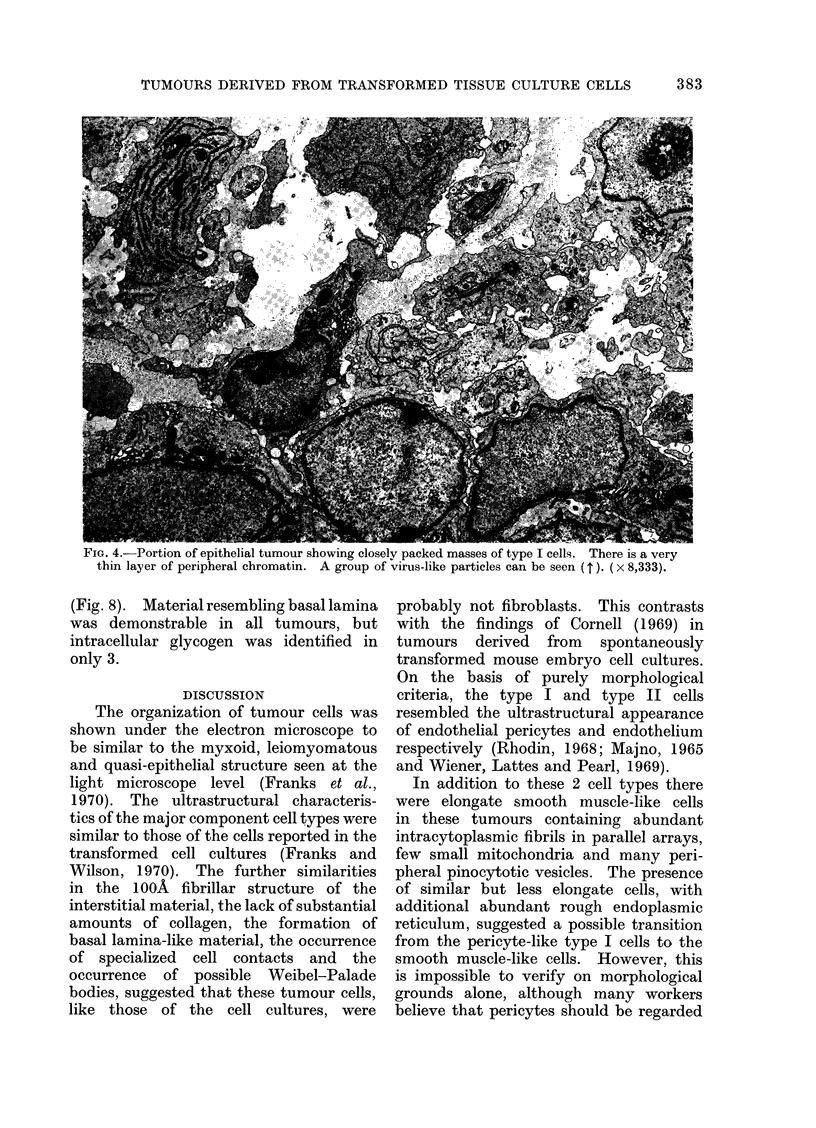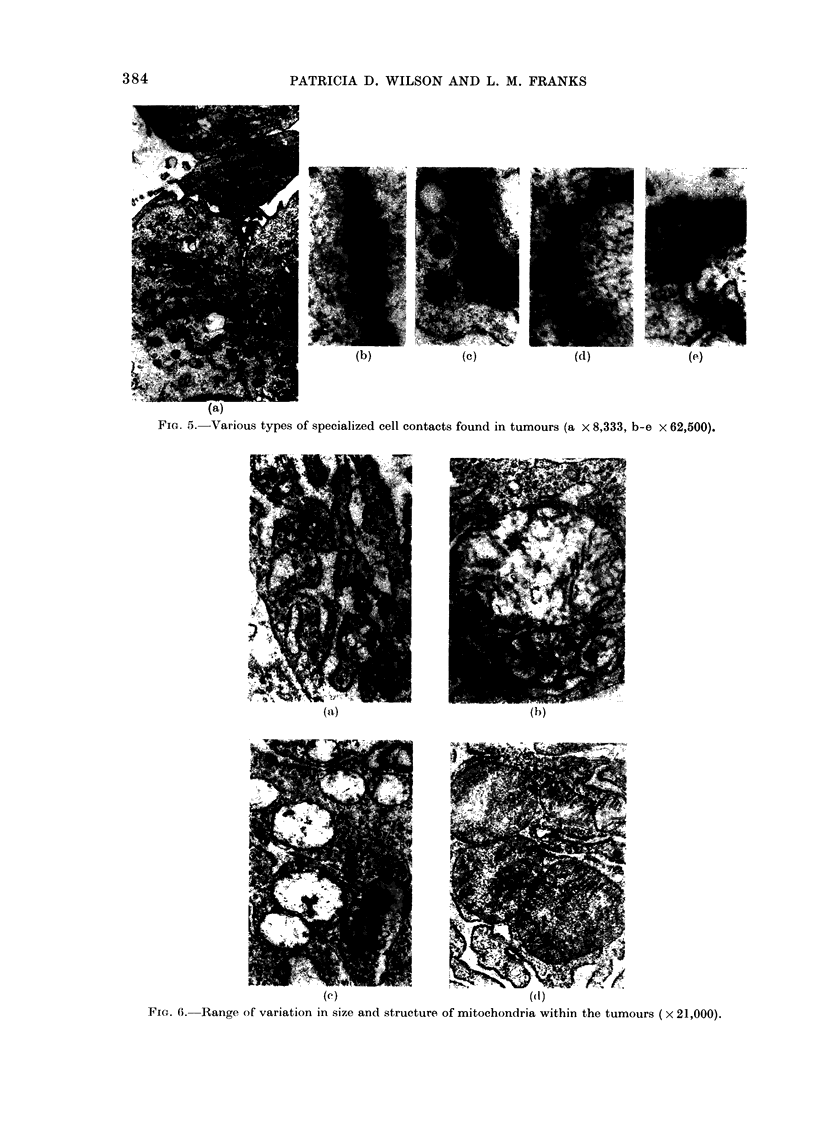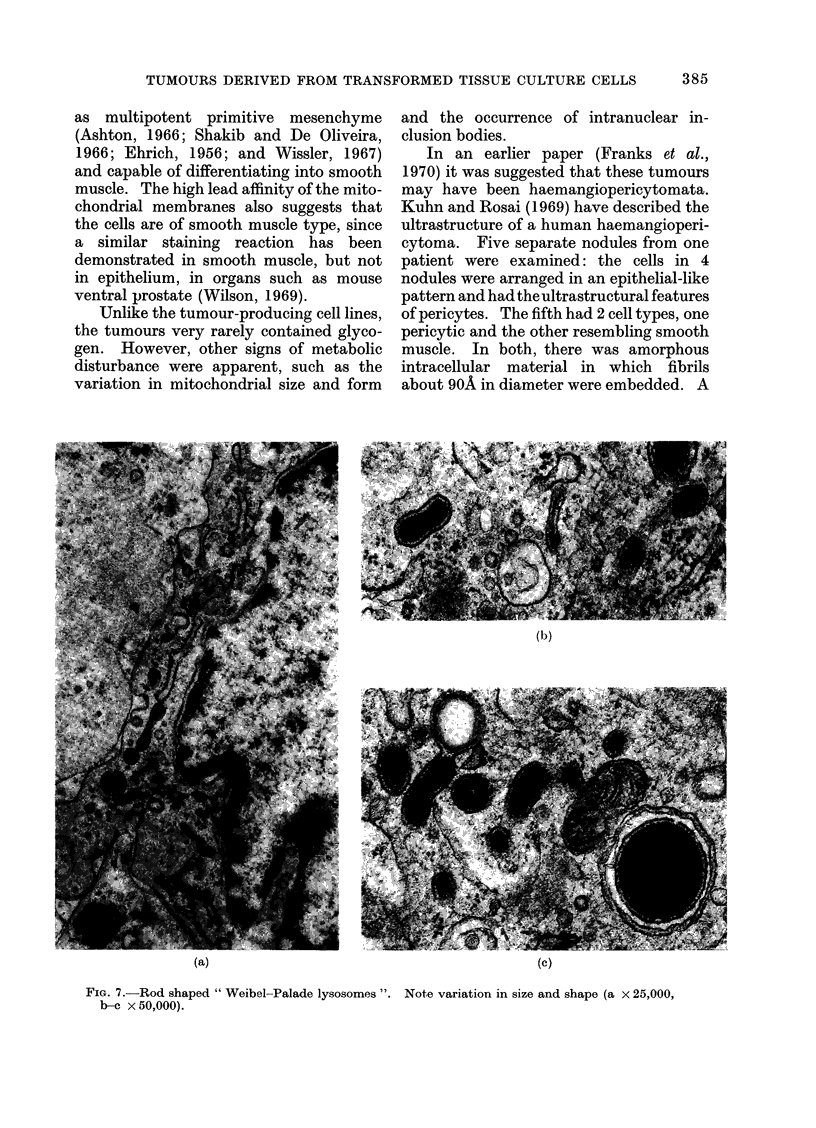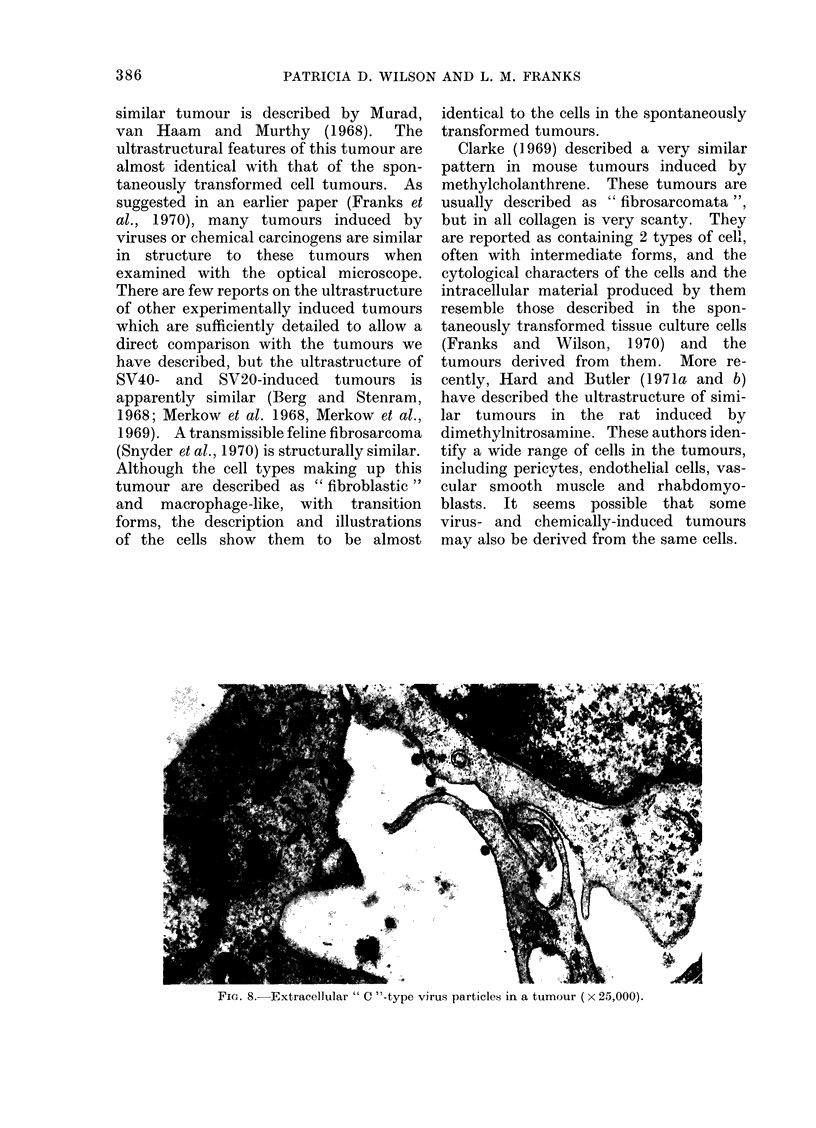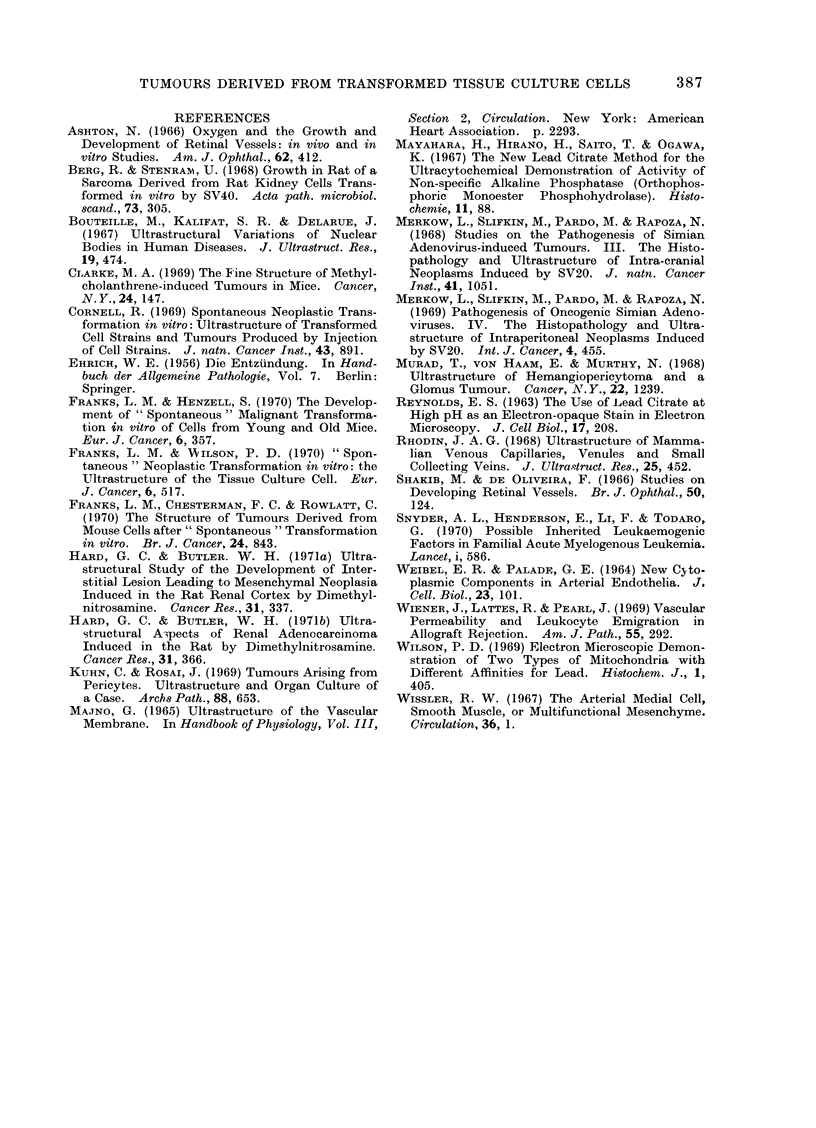# The Ultrastructure of Tumours Derived from Spontaneously Transformed Tissue Culture Cells

**DOI:** 10.1038/bjc.1972.50

**Published:** 1972-10

**Authors:** Patricia D. Wilson, L. M. Franks

## Abstract

**Images:**


Br. J. Cancer (1972) 26, 380

### THE ULTRASTRUCTURE OF TUMOURS DERIVED FROM SPONTANEOUSLY TRANSFORMED TISSUE CULTURE CELLS

PATRICIA D. WILSON AND L. M. FRANKS

*From the Department of Cellular Pathology, Imperial Cancer Research Fund, Lincoln's Inn Fields, London W.C.2*

Received 1 May 1972. Accepted 22 May 1972

**Summary.—The ultrastructure of 16 tumours derived from spontaneously transformed cell lines established from young and old C57 and C3H mouse organs is described. Three types of tumour were found: myxoid (fibrosarcomatous), consisting of cells with long processes and much interstitial material; leiomyomatous, consisting of a bundle of smooth muscle-like cells with less interstitial material; or epithelial-like consisting of closely packed round cells with little interstitial material. The cell types in the tumours were similar to those found in the tissue culture cell lines from which they were derived.**

IN earlier papers the establishment of neoplastic and non-neoplastic cell lines from many different organs of young and old C3H and C57 mice (Franks and Henzell, 1970) has been reported, as has the ultrastructure of the cells (Franks and Wilson, 1970). The light microscope morphology of tumours produced by implantation of some of those cell lines into syngeneic mice showed a mixed pattern with myxoid, leiomyomatous and pseudo-epithelial areas (Franks, Chesterman and Rowlatt, 1970). Few studies have been made of the ultrastructure of tumours derived from cells spontaneously transformed *in vitro* but Cornell (1969) concluded that the cells of tumours originating from transformed murine embryo cell strains were fibroblastic. The present paper describes the ultrastructure of the cell types and their arrangement in the tumours.

### MATERIALS AND METHODS

Tumour-producing cell lines were established from young (3–20 days) and old (28–34 months) C57 BLa$^{t}$Icrf and C3H mouse kidney, lung, bladder, heart, prostate gland, tongue, spinal cord, nerve and brain (Franks and Henzell, 1970). On subcutaneous injection of approximately $3 \times 10^6$ cells into syngeneic 3–6 months old mice, primary tumours were established which were then transplanted or stored in liquid nitrogen (Franks *et al.*, 1970). Tissues from 16 tumours (6 derived from young, and 10 from old mice) were sliced and fixed for 4 hours in 2·5% glutaraldehyde in 0·1 mol/l sodium cacodylate buffer at 4° C, rinsed overnight in 0·1 mol/l sodium cacodylate buffer at 4° C and post fixed in Palade's fluid for 1 hour over ice. Tissue blocks were dehydrated in graded ethanol, stained with 5% uranyl acetate in absolute alcohol and embedded in Araldite using epoxypropane as transitional solvent. Ultrathin sections were cut on a Sorvall MT2 ultramicrotome, stained with lead citrate (Reynolds, 1963) and viewed in an Hitachi HS7S or Siemens Elmiskop 1 electron microscope. Some tissues were processed for the electron microscopic demonstration of alkaline phosphatase by the methods of Mayahara *et al.* (1967).

### RESULTS

Basically similar types of cell were found in all 16 tumours, but there were differences in organization of the cells and in quantities of interstitial material present.

The cell types found were similar to the type I, type II and intermediate cells previously described in the cell cultures (Franks and Wilson, 1970). Type I cells typically contained a large, round or bean-shaped nucleus with a single nucleolus and a very thin layer of chromatin condensed against the nuclear membrane. The cytoplasm contained rough endoplasmic reticulum, many free ribosomes, a Golgi zone but relatively few mitochondria, lysosomes or autophagic vacuoles. Type II cells typically contained a convoluted nucleus with one or more prominent nucleoli, and more chromatin than in type I cells in a thick peripheral layer and in clumps. The cytoplasm often contained distended rough endoplasmic reticulum, a large Golgi zone and many ribosomes, mitochondria, lysosomes and autophagic vacuoles. Intermediates between the 2 cell types were also found.

In these tumours there were frequently some cells present which resembled smooth muscle cells. These were elongated cells which contained many cytoplasmic fibrils in parallel arrays, small mitochondria and many peripheral pinocytotic vesicles. Occasional giant cells were found which resembled type I or type II cells. Most tumours contained a few eosinophils, polymorphs, basophils, neutrophils, monocytes and lymphocytes.

The organization of tumour cells fell into 3 categories, corresponding with the myxoid (fibrosarcomatous), leiomyomatous and epithelial-like structure found at the light microscope level (Franks *et al.*, 1970). The myxoid type showed a relatively loose, open arrangement of elongate cells with long processes. Type II cells were predominant and a considerable amount of interstitial material was present (Fig. 1). The leiomyomatous type was typically composed of very elongated cells resembling smooth muscle and arranged in bands. These cells contained many microfibrils and were found in a tighter arrangement, with less interstitial material present than in the myxoid type (Fig. 2, 3). The epithelial type of tumour was composed of closely packed, rounded cells; little interstitial material was present. Type I cells were predominant (Fig. 4). Some tumours contained regions of all 3 patterns. Tumour blood vessels were also frequently seen.

The structure of the extracellular, interstitial material, particularly abundant in the myxoid tumour types, was similar to that in the cell cultures, *i.e.*, an amorphous material and fine fibrils of approximately 100Å diameter with an electron dense outer rim and lucent core (Franks and Wilson, 1970).

Specialized cell contacts were frequently found between similar and dissimilar cell types. Most of these resembled the intermediate junctions (zonula adherens) of epithelial cells, although atypical tight junctions (zonula occludens) were occasionally seen. These contacts were often hazy in appearance and thus difficult to classify (Fig. 5).

Mitochondria exhibited great variation within the tumours. Unlike the " dense " mitochondria in the cell cultures, the matrix was always electron lucent in tumour mitochondria. There was, however, enormous variation in size, shape and cristal configuration (Fig. 6). Some were obviously degenerating, showing swelling and reduction of cristae. Others were Y-shaped or indented. The mitochondria of all type I, type II and smooth muscle-like cells within the spontaneously transformed tumours showed a high affinity for lead when incubated in media for the demonstration of alkaline phosphatase, which contained high lead ion concentration. A similar reaction was seen in the mitochondria of tumour blood vessel endothelium and pericytes.

Rod-shaped " lysosomes ", not unlike those described by Weibel and Palade (1964) in human endothelial cells, were found in all 16 tumours, predominantly in type II cells. However, there was variation in their size and shape (Fig. 7).

Intranuclear inclusions of types 1–4 in Bouteille's (1967) classification, lamellar figures and cytoplasmic inclusions were frequently present. Virus-like particles (C-type) were identified in only 4 tumours

FIG. 1.—Portion of myxoid tumour showing numerous cell processes and abundant interstitial tissue. There is a blood vessel below and a type II tumour cell bottom left with a thick marginal zone of peripheral chromatin. The nucleus is less convoluted than usual. Note the resemblance to the endothelial cell (× 8,333).

FIG. 2.—Portion of leiomyomatous tumour showing closely packed bundles of cells (× 21,000).

FIG. 3.—Portion of similar tumour. The cytoplasm is packed with bundles of filaments (× 21,000).

FIG. 4.—Portion of epithelial tumour showing closely packed masses of type I cells. There is a very thin layer of peripheral chromatin. A group of virus-like particles can be seen (↑). (×8,333).

(Fig. 8). Material resembling basal lamina was demonstrable in all tumours, but intracellular glycogen was identified in only 3.

### DISCUSSION

The organization of tumour cells was shown under the electron microscope to be similar to the myxoid, leiomyomatous and quasi-epithelial structure seen at the light microscope level (Franks *et al.*, 1970). The ultrastructural characteristics of the major component cell types were similar to those of the cells reported in the transformed cell cultures (Franks and Wilson, 1970). The further similarities in the 100Å fibrillar structure of the interstitial material, the lack of substantial amounts of collagen, the formation of basal lamina-like material, the occurrence of specialized cell contacts and the occurrence of possible Weibel–Palade bodies, suggested that these tumour cells, like those of the cell cultures, were probably not fibroblasts. This contrasts with the findings of Cornell (1969) in tumours derived from spontaneously transformed mouse embryo cell cultures. On the basis of purely morphological criteria, the type I and type II cells resembled the ultrastructural appearance of endothelial pericytes and endothelium respectively (Rhodin, 1968; Majno, 1965 and Wiener, Lattes and Pearl, 1969).

In addition to these 2 cell types there were elongate smooth muscle-like cells in these tumours containing abundant intracytoplasmic fibrils in parallel arrays, few small mitochondria and many peripheral pinocytotic vesicles. The presence of similar but less elongate cells, with additional abundant rough endoplasmic reticulum, suggested a possible transition from the pericyte-like type I cells to the smooth muscle-like cells. However, this is impossible to verify on morphological grounds alone, although many workers believe that pericytes should be regarded

FIG. 5.—Various types of specialized cell contacts found in tumours (a ×8,333, b-e ×62,500).

FIG. 6.—Range of variation in size and structure of mitochondria within the tumours (×21,000).

as multipotent primitive mesenchyme (Ashton, 1966; Shakib and De Oliveira, 1966; Ehrich, 1956; and Wissler, 1967) and capable of differentiating into smooth muscle. The high lead affinity of the mitochondrial membranes also suggests that the cells are of smooth muscle type, since a similar staining reaction has been demonstrated in smooth muscle, but not in epithelium, in organs such as mouse ventral prostate (Wilson, 1969).

Unlike the tumour-producing cell lines, the tumours very rarely contained glycogen. However, other signs of metabolic disturbance were apparent, such as the variation in mitochondrial size and form and the occurrence of intranuclear inclusion bodies.

In an earlier paper (Franks *et al.*, 1970) it was suggested that these tumours may have been haemangiopericytomata. Kuhn and Rosai (1969) have described the ultrastructure of a human haemangiopericytoma. Five separate nodules from one patient were examined: the cells in 4 nodules were arranged in an epithelial-like pattern and had the ultrastructural features of pericytes. The fifth had 2 cell types, one pericytic and the other resembling smooth muscle. In both, there was amorphous intracellular material in which fibrils about 90Å in diameter were embedded. A

FIG. 7.—Rod shaped " Weibel–Palade lysosomes ". Note variation in size and shape (a ×25,000, b–c ×50,000).

similar tumour is described by Murad, van Haam and Murthy (1968). The ultrastructural features of this tumour are almost identical with that of the spontaneously transformed cell tumours. As suggested in an earlier paper (Franks *et al.*, 1970), many tumours induced by viruses or chemical carcinogens are similar in structure to these tumours when examined with the optical microscope. There are few reports on the ultrastructure of other experimentally induced tumours which are sufficiently detailed to allow a direct comparison with the tumours we have described, but the ultrastructure of SV40- and SV20-induced tumours is apparently similar (Berg and Stenram, 1968; Merkow *et al.* 1968, Merkow *et al.*, 1969). A transmissible feline fibrosarcoma (Snyder *et al.*, 1970) is structurally similar. Although the cell types making up this tumour are described as " fibroblastic " and macrophage-like, with transition forms, the description and illustrations of the cells show them to be almost identical to the cells in the spontaneously transformed tumours.

Clarke (1969) described a very similar pattern in mouse tumours induced by methylcholanthrene. These tumours are usually described as " fibrosarcomata ", but in all collagen is very scanty. They are reported as containing 2 types of cell, often with intermediate forms, and the cytological characters of the cells and the intracellular material produced by them resemble those described in the spontaneously transformed tissue culture cells (Franks and Wilson, 1970) and the tumours derived from them. More recently, Hard and Butler (1971*a* and *b*) have described the ultrastructure of similar tumours in the rat induced by dimethylnitrosamine. These authors identify a wide range of cells in the tumours, including pericytes, endothelial cells, vascular smooth muscle and rhabdomyoblasts. It seems possible that some virus- and chemically-induced tumours may also be derived from the same cells.

FIG. 8.—Extracellular " C "-type virus particles in a tumour ($\times$ 25,000).

### REFERENCES

ASHTON, N. (1966) Oxygen and the Growth and Development of Retinal Vessels: *in vivo* and *in vitro* Studies. *Am. J. Ophthal.*, **62**, 412.

BERG, R. & STENRAM, U. (1968) Growth in Rat of a Sarcoma Derived from Rat Kidney Cells Transformed *in vitro* by SV40. *Acta path. microbiol. scand.*, **73**, 305.

BOUTEILLE, M., KALIFAT, S. R. & DELARUE, J. (1967) Ultrastructural Variations of Nuclear Bodies in Human Diseases. *J. Ultrastruct. Res.*, **19**, 474.

CLARKE, M. A. (1969) The Fine Structure of Methylcholanthrene-induced Tumours in Mice. *Cancer, N.Y.*, **24**, 147.

CORNELL, R. (1969) Spontaneous Neoplastic Transformation *in vitro*: Ultrastructure of Transformed Cell Strains and Tumours Produced by Injection of Cell Strains. *J. natn. Cancer Inst.*, **43**, 891.

EHRICH, W. E. (1956) Die Entzündung. In *Handbuch der Allgemeine Pathologie*, Vol. 7. Berlin: Springer.

FRANKS, L. M. & HENZELL, S. (1970) The Development of " Spontaneous " Malignant Transformation *in vitro* of Cells from Young and Old Mice. *Eur. J. Cancer*, **6**, 357.

FRANKS, L. M. & WILSON, P. D. (1970) " Spontaneous " Neoplastic Transformation *in vitro*: the Ultrastructure of the Tissue Culture Cell. *Eur. J. Cancer*, **6**, 517.

FRANKS, L. M., CHESTERMAN, F. C. & ROWLATT, C. (1970) The Structure of Tumours Derived from Mouse Cells after " Spontaneous " Transformation *in vitro*. *Br. J. Cancer*, **24**, 843.

HARD, G. C. & BUTLER, W. H. (1971*a*) Ultrastructural Study of the Development of Interstitial Lesion Leading to Mesenchymal Neoplasia Induced in the Rat Renal Cortex by Dimethylnitrosamine. *Cancer Res.*, **31**, 337.

HARD, G. C. & BUTLER, W. H. (1971*b*) Ultrastructural Aspects of Renal Adenocarcinoma Induced in the Rat by Dimethylnitrosamine. *Cancer Res.*, **31**, 366.

KUHN, C. & ROSAI, J. (1969) Tumours Arising from Pericytes. Ultrastructure and Organ Culture of a Case. *Archs Path.*, **88**, 653.

MAJNO, G. (1965) Ultrastructure of the Vascular Membrane. In *Handbook of Physiology, Vol. III, Section 2, Circulation*. New York: American Heart Association. p. 2293.

MAYAHARA, H., HIRANO, H., SAITO, T. & OGAWA, K. (1967) The New Lead Citrate Method for the Ultracytochemical Demonstration of Activity of Non-specific Alkaline Phosphatase (Orthophosphoric Monoester Phosphohydrolase). *Histochemie*, **11**, 88.

MERKOW, L., SLIFKIN, M., PARDO, M. & RAPOZA, N. (1968) Studies on the Pathogenesis of Simian Adenovirus-induced Tumours. III. The Histopathology and Ultrastructure of Intra-cranial Neoplasms Induced by SV20. *J. natn. Cancer Inst.*, **41**, 1051.

MERKOW, L., SLIFKIN, M., PARDO, M. & RAPOZA, N. (1969) Pathogenesis of Oncogenic Simian Adenoviruses. IV. The Histopathology and Ultrastructure of Intraperitoneal Neoplasms Induced by SV20. *Int. J. Cancer*, **4**, 455.

MURAD, T., VON HAAM, E. & MURTHY, N. (1968) Ultrastructure of Hemangiopericytoma and a Glomus Tumour. *Cancer, N.Y.*, **22**, 1239.

REYNOLDS, E. S. (1963) The Use of Lead Citrate at High pH as an Electron-opaque Stain in Electron Microscopy. *J. Cell Biol.*, **17**, 208.

RHODIN, J. A. G. (1968) Ultrastructure of Mammalian Venous Capillaries, Venules and Small Collecting Veins. *J. Ultrastruct. Res.*, **25**, 452.

SHAKIB, M. & DE OLIVEIRA, F. (1966) Studies on Developing Retinal Vessels. *Br. J. Ophthal.*, **50**, 124.

SNYDER, A. L., HENDERSON, E., LI, F. & TODARO, G. (1970) Possible Inherited Leukaemogenic Factors in Familial Acute Myelogenous Leukemia. *Lancet*, i, 586.

WEIBEL, E. R. & PALADE, G. E. (1964) New Cytoplasmic Components in Arterial Endothelia. *J. Cell. Biol.*, **23**, 101.

WIENER, J., LATTES, R. & PEARL, J. (1969) Vascular Permeability and Leukocyte Emigration in Allograft Rejection. *Am. J. Path.*, **55**, 292.

WILSON, P. D. (1969) Electron Microscopic Demonstration of Two Types of Mitochondria with Different Affinities for Lead. *Histochem. J.*, **1**, 405.

WISSLER, R. W. (1967) The Arterial Medial Cell, Smooth Muscle, or Multifunctional Mesenchyme. *Circulation*, **36**, 1.